# A Case Series of Stereotactic Biopsy of Brainstem Lesions through the Transfrontal Approach

**DOI:** 10.1055/s-0042-1758696

**Published:** 2022-11-25

**Authors:** Oscar Andrés Escobar-Vidarte, Dylan Paul Griswold, Javier Orozco-Mera, Juan Felipe Mier-Garcia, Fernando Peralta Pizza

**Affiliations:** 1 Department of Neurosurgery, University del Valle, Cali, Valle del Cauca, Colombia; 2 Department of Neurosurgery, University Hospital del Valle, Cali, Valle del Cauca, Colombia; 3 Department of Neurosurgery, Castellana Clinic, Cali, Valle del Cauca, Colombia; 4 Department of Neurosurgery, Latin American Institute of Neurology and the Nervous System, Bogota, Colombia; 5 Department of Clinical Neurosciences, University of Cambridge, Cambridge, Cambridgeshire, United Kingdom; 6 School of Medicine, Stanford Medical School, Stanford, California, United States; 7 Department of Neurosurgery, University Hospital Tomas Uribe Uribe of Tuluá, Tuluá, Valle del Cauca Colombia

**Keywords:** brainstem biopsy, stereotactic surgery, transfrontal approach, brain tumor

## Abstract

**Background and Importance** Brainstem lesions may be unresectable or unapproachable. Regardless, the histopathological diagnosis is fundamental to determine the most appropriate treatment. We present our experience with transfrontal stereotactic biopsy technique for brainstem lesions as a safe and effective surgical route even when contralateral transhemispheric approach is required for preservation of eloquent tissue.

**Clinical Presentation** Twenty-five patients underwent surgery by transfrontal approach. Medical records were reviewed for establishing the number of patients who had postoperative histopathological diagnosis and postoperative complications. Twenty-four patients (18 adults and 7 children) had histopathological diagnosis. There were 18 astrocytomas documented, of which 12 were high grade and 6 low grade. The other diagnoses included viral encephalitis, post–renal transplant lymphoproliferative disorder, nonspecific chronic inflammation, Langerhans cell histiocytosis, and two metastases. No case was hindered by cerebrospinal fluid loss or ventricular entry. Complications included a case of mesencephalic hemorrhage with upper limb monoparesis and a case of a partially compromised third cranial nerve in another patient without associated bleeding.

**Conclusion** Stereotactic biopsy of brainstem lesions by transfrontal ipsilateral or transfrontal transhemispheric contralateral approaches is a safe and effective surgical approach in achieving a histopathological diagnosis in both pediatric and adult populations.

## Introduction

Tumors of the brainstem correspond to approximately 1.6% of all tumors of the central nervous system and 10 to 15% of all intracranial tumors in the pediatric population. 1 2 The brainstem contains a critically important, life-sustaining ascending and descending fiber system. This severely limits the resectability of lesions in this location. However, histopathological, immunohistochemical, genetic, and molecular diagnosis of brainstem lesions guides clinicians in their ultimate diagnosis and subsequent treatment plan.

In this context, the need arises for a safe and effective surgical technique to obtain an adequate amount of tissue while preserving eloquent areas. Pure radiological findings will often fail to correctly diagnose brainstem lesions, as magnetic resonance imaging (MRI)-based diagnosis has been reported as high as 10 to 20% and MRI-based classification and grading was estimated to be correct in 35% of low-grade gliomas and 27% of high-grade gliomas. 3 4 5 Stereotactic-guided biopsy (STB) has been used for this purpose, evolving alongside new imaging devices and stereotactic planning software. 6 7 Patient-specific anatomical mapping has the capability of creating three-dimensional objects, including critical structures of the brainstem. Synthetic tissue models can be applied to classify brain tissues in order to detect abnormalities. This tissue-based automatic segmentation results in highly individualized patient datasets for reliable extraction of deep brain stimulation targets. 8 9 Here, we present our experience with STB of brainstem lesions by transfrontal route, even when contralateral transhemispheric approach is required for preservation of eloquent tissue.

## Case Report

Between 2013 and 2020, 25 patients with unresectable brainstem lesions were selected for STB to determine the histopathological diagnosis.

The patients underwent preoperative brain MRI (axial sections, 2 mm, T1 sequences with contrast medium and T2). On the day of surgery, pediatric patients underwent general anesthesia and adult patients underwent sedation. Zamorano-Duchovny (Inomed, Emmendingen, Germany) or Riechert-Mundinger (Inomed, Emmendingen, Germany) stereotactic frames were then positioned. Subsequently, a contrast-enhanced brain tomography was obtained with axial sections of 2 mm under stereotactic conditions. In Praezis Plus 3.0 (Tratamed, Slovak Republic) or IPS 4.0, 5.0 or 6.0 (Inomed, Emmendingen, Germany) high-precision stereotactic planning software, image fusion between resonance and tomography was performed to plan the trajectory of the biopsy needle from a precoronal or coronal and ipsilateral paramedian entry point to the lesion. Ipsilateral or contralateral routes were traced in order to maximally preserve the ventricles and the arterial and venous vascular structures; ipsilateral routes were generally preferred; nonetheless, should the aimed trajectory include or violate unequivocally the ventricles, basal cisterns, or any blood vessel within them, a contralateral approach was elected. The needle was then inserted through a frontal trephine hole, and following the planned trajectory, tissue samples from four quadrants of the lesion were acquired. After the sample was taken, the needle was gently withdrawn, and the surgical procedure completed.

**Fig. 1:**
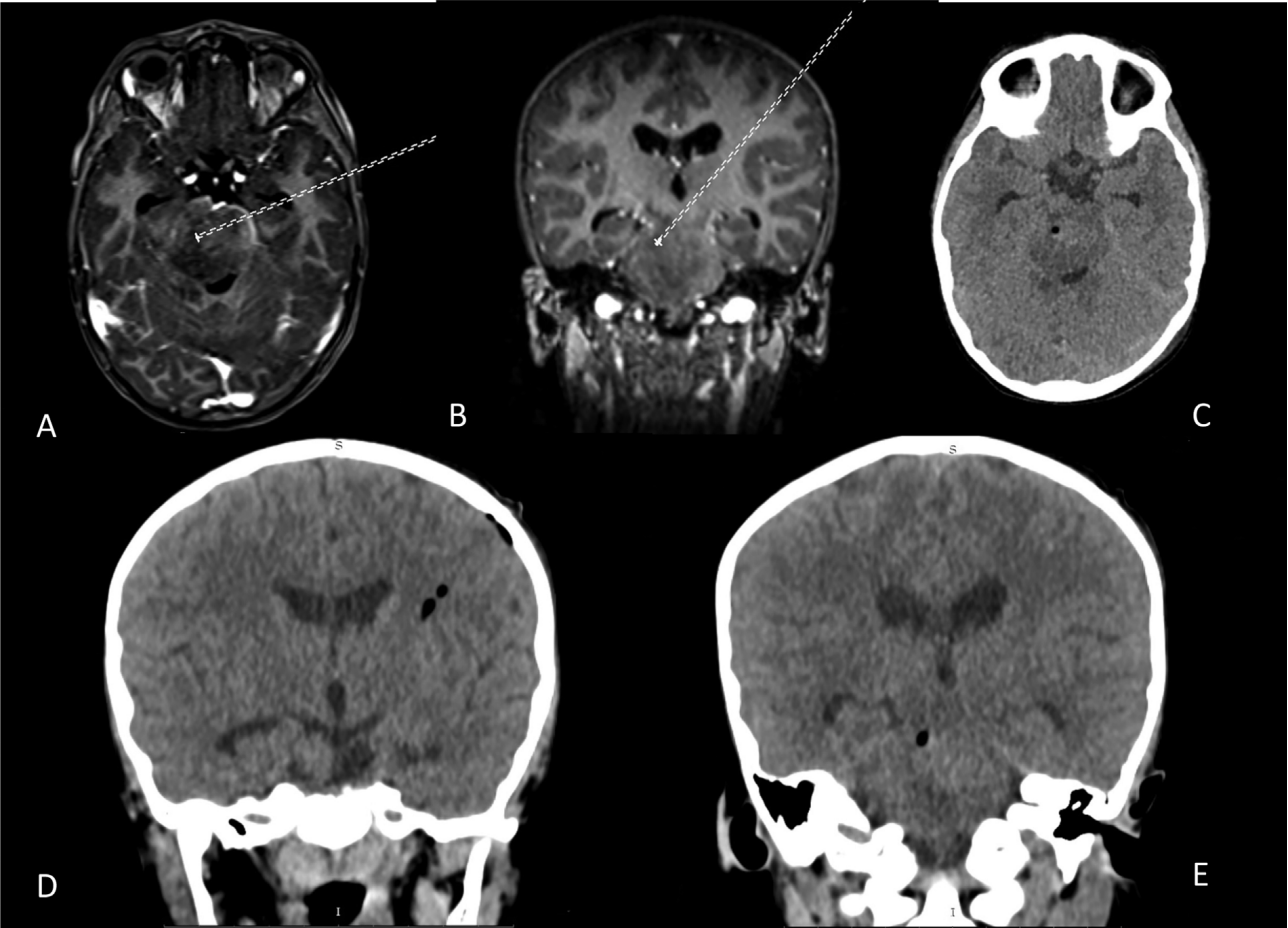
Preoperative brain magnetic resonance imaging axial view ( **A** ) and coronal view ( **B** ) showing a planned transhemispheric trajectory from a left frontal entry point to a right intra-axial mesencephalic lesion. Postoperative brain tomography axial view ( **C** ) and coronal view ( **D, E** ) at the site of biopsy sampling.

There were 7 pediatric patients and 18 adult patients with an average age of 30.4 years (3 to 67 years); 13 patients were male and 12 were female. The transfrontal surgical approach was used in all cases ( Table 1 ). A transfrontal transhemispheric approach was taken in three patients with paramedian lesions at risk of vascular injury if an ipsilateral approach through the perimesencephalic cisterns was taken ( Fig. 1A–C ).

**Table 1:** General characteristics of the series of 25 patients with brainstem lesions undergoing stereotactic-guided biopsy

Case	Age (y)	Sex	Population	Location	Diagnosis	Complications	Length of stay	Pre-op deficits	Post-op condition	Ipsilateral or contralateral	Pre-op imaging
1	28	Female	Adult	Pons	Chronic demyelinating inflammatory disease	None	2 d	Quadriparesis	No change	Ipsilateral	MRI
2	9	Female	Pediatric	Bulbopontine	Posttransplant lymphoproliferative disorder	None	2 d	Bilateral cranial nerve VI	No change	Ipsilateral	MRI
3	32	Female	Adult	Pons	Astrocytoma WHO grade II	None	2 d	Quadriparesis	No change	Ipsilateral	MRI
4	41	Male	Adult	Mesencephalon	Astrocytoma WHO grade II	None	2 d	Hemiparesis, cranial nerve III	No change	Ipsilateral	MRI
5	6	Male	Pediatric	Mesencephalon	None	None	2 d	Parinaud syndrome	No change	Ipsilateral	MRI
6	3	Female	Pediatric	Bulbopontine	Astrocytoma WHO grade IV	None	2 wk	Quadriparesis, cranial nerves VI, VII, IX, X, and XII	No change	Ipsilateral	MRI
7	5	Female	Pediatric	Bulbopontine	Viral encephalitis	None	1 mo	Quadriparesis, cranial nerves VI and VII	No change	Ipsilateral	MRI
8	58	Female	Adult	Mesencephalon	Astrocytoma WHO grade IV	None	3 wk	Hemiparesis, cranial nerves III and IV	No change	Ipsilateral	MRI
9	33	Male	Adult	Mesencephalon	Astrocytoma WHO grade III	None	2 wk	Hemiparesis	No change	Ipsilateral	MRI
10	8	Male	Pediatric	Pons	Astrocytoma WHO grade III	None	2 wk	Hemiparesis, cranial nerves VI and VII	No change	Contralateral	MRI
11	12	Male	Pediatric	Pons	Astrocytoma WHO grade II	None	2 d	None	No change	Ipsilateral	MRI
12	67	Female	Adult	Mesencephalon	Metastasis	None	1 wk	Hemiparesis, bilateral cranial nerve VI	No change	Ipsilateral	MRI
13	51	Female	Adult	Pons	Astrocytoma WHO grade III	None	2 wk	Quadriparesis, bilateral cranial nerve VI	No change	Ipsilateral	MRI
14	28	Female	Adult	Mesencephalon	Langerhans cell histiocytosis	None	2 d	None	No change	Ipsilateral	MRI
15	34	Female	Adult	Bulbopontine	Astrocytoma WHO grade II	Mesencephalic hemorrhage: upper limb monoparesis	5 d	None	Upper limb monoparesis	Ipsilateral	MRI
16	27	Female	Adult	Mesencephalon	Astrocytoma WHO grade II	None	2 d	None	No change	Ipsilateral	MRI
17	23	Male	Adult	Mesencephalon	Astrocytoma WHO grade IV	None	1 mo	Quadriparesis, Parinaud syndrome, bilateral cranial nerve VI	No change	Ipsilateral	MRI
18	45	Male	Adult	Mesencephalon	Metastasis	None	3 wk	Cranial nerves III and IV	No change	Ipsilateral	MRI
19	51	Female	Adult	Pons	Astrocytoma WHO grade III	None	2 wk	Quadriparesis, cranial nerve VI	No change	Contralateral	MRI
20	39	Male	Adult	Pons	Astrocytoma WHO grade III	None	1 wk	Quadriparesis	No change	Contralateral	MRI
21	41	Male	Adult	Mesencephalon	Astrocytoma WHO grade III	Transient cranial nerve III palsy	1 wk	Hemiparesis	Transient cranial nerve III palsy	Ipsilateral	MRI
22	19	Male	Adult	Mesencephalon	Astrocytoma WHO grade II	None	2 d	None	No change	Ipsilateral	MRI
23	43	Male	Adult	Mesencephalon	Astrocytoma WHO grade IV	None	1 wk	Parinaud syndrome	No change	Ipsilateral	MRI
24	52	Male	Adult	Bulbopontine	Astrocytoma WHO grade III	None	1 wk	Quadriparesis, cranial nerves VI, VII, VIII, IX, X, and XII	No change	Ipsilateral	MRI
25	7	Male	Pediatric	Pons	Astrocytoma WHO grade IV	None	5 d	Quadriparesis	No change	Ipsilateral	MRI

Abbreviations: MRI, magnetic resonance imaging; WHO, World Health Organization.

Eighteen patients were preoperatively diagnosed with a glioma tumor: 12 high-grade astrocytomas (World Health Organization [WHO] grade III and grade IV) and 6 low-grade astrocytomas (WHO grade I and grade II). Six patients had other histopathological diagnosis: one case of viral encephalitis, one case of posttransplant lymphoproliferative disorder, one case of chronic demyelinating inflammatory disease, one case of Langerhans cell histiocytosis, and two cases of metastasis ( Table 1 ). One patient did not have a histopathological diagnosis. In total, a definitive diagnosis was achieved in 96% of the cases.

The postoperative complication rate was 8%. In the immediate postoperative period, a case of mesencephalic hemorrhage associated with right upper extremity monoparesis with partial improvement during follow-up was documented. One patient experienced transient left third cranial nerve palsy without associated radiologic bleeding, which resolved with spontaneous complete recovery. No case was hindered by cerebrospinal fluid (CSF) loss or ventricular entry. There were no documented deaths associated with the procedures.

Length of stay was greatly variable, as many patients were receiving oncological treatment or other medical treatments that depended upon the biopsy result. Furthermore, the neurological condition of others did not allow for a safe discharge home. Only two patients with neurological deficits secondary to the procedure prolonged their hospitalization time. Lastly, all patients underwent MRI without tractography, as the technology is not yet available at our hospital.

## Discussion

Biopsies of brainstem lesions can be a constant challenge even for the most experienced of neurosurgeons. There is always a significant risk of neurological deterioration and catastrophic bleeding. Additionally, the surgeon must also ensure enough sample is obtained. Our experience shows that transfrontal STB for brainstem lesions can be, with a meticulous planning process and performance, a safe and reproducible procedure capable of obtaining the necessary tissue samples with an acceptable accuracy. Stereotactic-guided biopsies of brainstem lesions have reduced the morbidity and mortality rates of those seen with brainstem biopsy via craniotomy. 6 Its use has become widespread, and despite progress in state-of-the-art imaging techniques, imaging diagnosis is far from the gold standard method of histopathology. 4 7

In allowing a definitive histopathological diagnosis for complex lesions to be made, the optimal treatment plan can be tailored to both adult and pediatric patients with a low probability of neurological status deterioration. The transfrontal approach is also versatile as it allows the surgeon to access contralateral lesions. Creating access to brainstem lesions through this route permits the surgeon to avoid the transcerebellar approach, which creates unnatural patient positioning, anesthetic complexity, and difficulty in approaching skin and deep tissues. 10 11 Furthermore, the positioning and manipulation of stereotactic devices is compromised, leading to increased risk of complications and failures in obtaining adequate tissue sample. 12

In a study of 142 patients submitted to stereotactic biopsy of the brainstem through either the suboccipital transcerebellar and the transfrontal approach, it was found that the diagnosis rate in the transcerebellar approach was 84.2 and 95.1% for patients biopsied via the transfrontal trajectory. 13 Other studies have shown that both the transfrontal and transcerebellar routes do not have significant difference in complication rates, nor diagnostic accuracy. 14 15 16 17 18 For midbrain lesions, it is suggested that a supratentorial transfrontal approach may be better, while the transcerebellar–transpeduncular trajectory may be better suited for pontine lesions that come along with a shorter trajectory length, thus decreasing the risk of bleeding or risk of induced microlesion in that eloquent area. 5 With minimal data directly comparing the two approaches, a larger prospective study with adequate sample size or a retrospective case–control study of similar lesions targeted by the two approaches is needed to better elucidate the pros and cons of each approach.

Transfrontal STB is considered a safe procedure, with high rates of diagnosis and low rates of complications, with hemorrhage at the sampling site the most commonly reported complication. 17 19 20 21 22 23 24 25 The series with the greatest epidemiological power reported diagnostic accuracy in 95 to 98% of cases, and a meta-analysis with 1,480 cases reported a positive diagnostic probability of 96.2%, with a morbidity of 7.8% and mortality of 0.9% of cases. 1 10 26 27 28 Its diagnostic efficacy has not been surpassed by modern imaging, which still does not provide enough information to establish prognosis and guide clinical therapeutic decision making. 14 15 28

It is important to note that while this is our center's experience, our case report is neither a randomized clinical trial nor a comparative study. Thus, the results presented here cannot be used to make generalized nor evidence-based recommendations, advocating for one technique and approach over another. However, this case report adds to the growing body of evidence that will help clarify the pros and cons of the transfrontal approach for stereotactic biopsy of the brainstem and may be used in a future systematic or narrative review.

## Conclusion

Our experience has shown that STB of brainstem lesions is an effective and safe procedure, capable of obtaining adequate sample volume needed to reach a definitive pathological diagnosis that can best guide therapeutic decision-making. The transfrontal approach may be a route of lesser complexity to the brainstem and may provide greater postoperative safety, allowing the surgeon to approach both ipsilateral and contralateral lesions avoiding critical perimesencephalic vascular structures and the violation of structures that could lead to brain shift due to CSF loss.
